# Naturalistic Topography Assessment in a Randomized Clinical Trial of Smoking Unfiltered Cigarettes: Challenges, Opportunities, and Recommendations

**DOI:** 10.3390/ijerph182211857

**Published:** 2021-11-12

**Authors:** Devan R. Romero, Kim Pulvers, Erika Carter, Casey Barber, Nora Satybaldiyeva, Thomas E. Novotny, Eyal Oren

**Affiliations:** 1 Department of Kinesiology and Psychology, California State University San Marcos, San Marcos, CA 92096, USA; kpulvers@csusm.edu (K.P.); carte075@cougars.csusm.edu (E.C.); 2 School of Public Health, University of Nevada, Las Vegas, NV 89154, USA; barberc8@unlv.nevada.edu; 3 School of Public Health, San Diego State University, San Diego, CA 91282, USA; ksatybaldiyeva3221@sdsu.edu (N.S.); tnovotny@sdsu.edu (T.E.N.); eoren@sdsu.edu (E.O.)

**Keywords:** nicotine, tobacco, topography, exposure, policy, environmental

## Abstract

Smoking topography (ST) is a set of measures profiling the behavioral characteristics of smoking in various settings. The CReSS portable device can measure ST in the natural environment. No standard protocol exists for measuring ST longitudinally with the CReSS. This study examined the utilization of the CReSS to measure ST and highlights challenges and opportunities in a naturalistic setting. This study is part of a randomized cross-over clinical trial of smoking filtered or unfiltered cigarettes. Participants (*n* = 43) smoked in each study condition for two weeks using the CReSS device for five days in their naturalistic smoking setting. The devices were calibrated and cleaned during the washout period, and data were downloaded every visit. Five test puffs were administered to calibrate each device. Moderate compliance rates (74.1%) were found with device usage, and the issues encountered were overheating/clogging, incorrectly registered date/time-stamped data, and device repair/replacement. Routine inspection/cleaning and training in device usage were instrumental in mitigating device malfunctioning. The CReSS device proved to be a feasible tool to examine naturalistic smoking topography and the potential impact of changes in tobacco product design on smoking unfiltered cigarettes. This is the first study to examine ST variables longitudinally, measured at multiple time points, and using unfiltered cigarettes.

## 1. Introduction

Smoking topography (ST) refers to a set of measures that describe smoking patterns in various settings [1,2,3]. These measures non-invasively reveal adaptations in smoking behaviors that can be associated with smoking biomarkers and carcinogenic exposures [2,3,4]. Typical ST measurements in laboratory settings are the number of puffs taken during a smoking session, puff volume (PV), puff duration (PD), inter-puff interval (IPI, or time between puffs), and peak flow rate (PFR, or airway function) [3,5]. One commonly used portable, battery-operated ST measuring device is the Clinical Research Support System (CReSS) pocket, manufactured by Borgwaldt Korber Solutions [3,5,6]. The CReSS functions as a flowmeter, measuring differential pressure to calculate puffing variables when smoking a tobacco product via a connected mouthpiece [3,5,7]. An important advantage of the CReSS device is that it can be used in direct clinical or laboratory-observed settings, or indirectly in the natural environment (e.g., at home) [1,8].

Smoking with a CReSS device provides valid and reliable measures of ST and toxicant exposures representative of smoking in a natural environment [2]. While a few studies have found greater enjoyment in and positive rewards from conventional smoking compared to utilizing the CReSS device [9,10], the direct and indirect observations of ST measurements do not differ based on a participant’s subjective use of the device [1,2,11]. Slightly higher measurement variability has been found in ST among individuals smoking with a CReSS device; however, many studies report consistent within-subject comparisons [2,8,11], supporting the CReSS device as a reliable tool for assessment of individual exposure and ST patterns. An initial challenge and crucial factor in measuring ST is calibrating the device to ensure topography standardization, both within and between participants, for all smoking sessions and conditions. Past studies used varying calibration standards for the CReSS device, mainly relying on manufacturer guidelines with added modifications and/or adaptations in directly observed laboratory or clinical settings [3,12]. However, detailed methods for troubleshooting CReSS device issues, such as calibration and other technical difficulties, are not available [13]. Most study designs were limited to one-to-six-time data points; most did not provide a longitudinal perspective with frequent ST assessments and had a short study timeframe of two weeks or less [8,9,10,14,15,16]. The few longitudinal studies had limited ST measurements: three over a 6-week period [17], and six over a 3-month period [18].

Although other studies evaluated smoking in naturalistic environments with laboratory verification, the methods and number of participants varied significantly or were not clearly reported. Despite the consensus that ST could be measured accurately even with variable smoking behavior and with measurement of exposure biomarkers, very few studies assessed participants longitudinally, and none evaluated unfiltered cigarettes [19]. 

We used the CReSS system to assess ST in a randomized trial of filtered vs. unfiltered cigarette smoking across multiple naturalistic settings. This paper describes the methodological challenges involved in using the portable CReSS device in this cross-over clinical trial. The results of this assessment will permit standardization of procedures and protocols to test for differences in smokers’ topography when switching between filtered and unfiltered cigarettes [20,21]. The smoking topography data, behavioral observations, and exposure differences are to be reported in separate manuscripts. 

## 2. Experimental Section

### Methods

**Figure 1:**
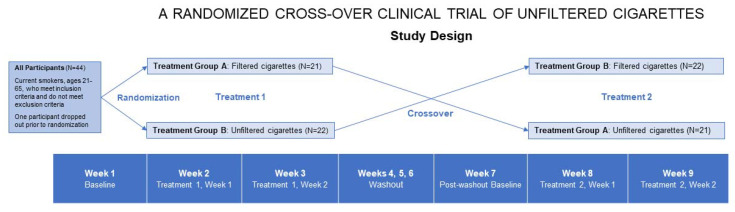
Randomized Cross-Over Clinical Trial of Unfiltered Cigarettes Study Design.

This study was a nine-week, randomized, cross-over clinical trial of unfiltered cigarettes that included a baseline visit at Week 1, a two-week treatment condition (Weeks 1 and 2), a three-week washout period between treatment conditions (Week 3), and a second baseline (post-washout) visit at Week 7 and concluded with a second two-week treatment condition (Weeks 8 and 9) (Figure 1). Participants (*n* = 43) were between the ages of 21 and 65 and met the criteria of being a “committed smoker,” meaning they had been smoking cigarettes for at least one year, were using filtered cigarettes (Camel and/or Pall Mall) at least two weeks before the study and smoked at least five cigarettes per day (CPD) on 25 days of the past month. Participants must have primarily smoked cigarettes (>50% of the time), allowing for secondary use of other tobacco products; however, women who were pregnant, nursing, or planning to get pregnant were excluded. Investigators were blinded as to the randomization of the treatment conditions (filtered and unfiltered cigarette smoking) (Table 1). Based on published research and the established half-life of primary tobacco biomarkers, we assumed three weeks to be a suitable timeframe for a “washout period” in order to eliminate the effect of exposures to the study cigarettes during the active treatment periods [21,22]. 

ST measures were captured on at least five days each week within daily eight-hour smoking periods. Both smoking conditions occurred in the participants’ naturalistic setting, and all smoking data were date/time stamped by the battery-operated CReSS pocket device (Borgwaldt-Hauni Korber Solutions, Hamburg, Germany). Of the 12 CReSS devices used in this study, three were purchased in 2015 for a pre-pilot study, and the other nine in 2019 from Borgwaldt Korber Solutions (n.d.) [6]. The CReSS device is unique in measuring ST and requires extensive instruction, demonstration, and follow-up for participants using the device. Participants were provided with in-lab training by research staff to demonstrate proper CReSS use, including how to verify the device was working (e.g., inserting the cigarette, observing LED color, and audible feedback), how to change/check batteries, and how to clean the mouthpiece. Instruction was given to clean the device at home with only a cotton swab and to not remove the mouthpiece or tubing from the device outside the lab. Participants were able to practice using the device during the initial visit, and each participant was provided with a reference guide, including study contact information, in case of problems or technical issues encountered at home. Lastly, participants followed the study protocol using the CReSS each visit, including calibrating the device by smoking a study cigarette in the lab and selecting device usage days (five days/week for a minimum of eight hours/day). Compensation was up to USD 695 for the entire study, including all lab visits, CReSS device usage, and returning smoked cigarette butts. The CReSS data were downloaded into an Access database during each visit, and participants returned the device for cleaning during the washout period (before the second treatment condition) and upon completion of the study. Research assistants used the manufacturer’s recommendations to clean the device and tubing. The complete study protocol is published elsewhere [22]. The study was approved by the San Diego State University Institutional Review Board (IRB) as human subjects research (Protocol #HS-2020-0071) and registered with ClinicalTrials.gov (NCT: 03749876).

## 3. Results 

### 3.1. Calibration and Standardization of CReSS Settings

To standardize the topography measurements, the CReSS device was calibrated at each visit by the research team. Using a syringe to inject ambient air, five test puffs were administered to calibrate each device and measure the volume and flow rate of the test puffs. The calibration involved (1) inserting an unlit cigarette directly into the orifice connected to the device tubing and syringe; (2) using the syringe to pull an “inhalation” at a medium pace to end with an abrupt stop at > 25 mL; (3) verifying the calculated volume with the CReSS software system; (4) using the volume reading from the syringe to develop a correction factor for the calibration settings. Each test puff had to sequentially fall within the ±2 mL range and have a PFR of 50–80 mL/s. If the puff volume was outside ±2 mL, then a scaling correction factor was used for our topography measurements. We found that one to three test puffs out of five were not consistently ±2 mL of 32 mL, nor was the peak flow rate constantly between 50 and 80 mL/s. Therefore, a conservative approach using five test puffs was adopted to ensure the reliability and validity of the device calibration. Over the course of the study, these inconsistencies lessened, possibly due to device training and experience with device troubleshooting by both staff and participants.

### 3.2. Compliance Rates

Compliance rates (CR) were evaluated based on the criterion of using the CReSS device on a minimum of five eight-hour days throughout the six weeks of the trial. We found a moderately high participant CR of 74.1% (*n* = 27 compliant), with non-compliance in 25.9% (*n* = 7) of participants observed as the study progressed and finalized after the three-week washout period. The highest number of days missed was one, most frequently occurring during the Week 7 (post-washout) and Weeks 8 and 9 (second two-week treatment condition) study visits (*n* = 3) (Figure 1). Four participants’ data were excluded due to missing data, six participants dropped out of the study, and data for seven participants could not be used due to COVID-19 restrictions and possession of the device outside the treatment condition timeframe (post-washout). These participants were excluded from the CR analysis. 

### 3.3. Other CReSS Device Issues 

This study employed 12 devices across 43 participants, with varying challenges across the participants. Specifically, technical issues were found in 9 of 12 devices used by 11 participants. Four devices suffered from overheating or clogging, requiring device repair or mouthpiece replacement. Five devices incorrectly registered date/time stamps. To address the complication posed by an incorrect date/time stamp that could affect data analysis, we synchronized the date/time with the computer at each calibration session during participant study visits and documented the device serial number. Due to device malfunctioning, five participants switched CReSS devices during treatment conditions.

### 3.4. Overheating and Clogging

Battery insertion with proper polarity alignment is essential to protect against overheating. Participants were instructed on battery insertion and daily cleaning of the orifice (where cigarettes are inserted), mouthpieces, and the tubing that attaches to the device. This was especially important because loose tobacco might be passed through the device with unfiltered cigarettes. Participants were provided with cleaning tools (e.g., cotton swabs), and the research team used additional methods during the washout period to clean and unclog the device as needed, especially after the unfiltered cigarette condition. Heavy tar buildup inside the tubes was observed and, at times, caused device malfunctioning with clogging and a reduction in proper airflow recording for the unfiltered cigarette condition. At two time points during the study, we sought assistance from the manufacturer to completely disassemble and properly clean the device with careful device reassembly. We did not anticipate the clogging problem in our study design, and the manufacturer did not provide any recommendations other than to disassemble and reassemble the devices after cleaning. After we identified the clogging issue, we reminded participants to frequently clean the mouthpiece with a cotton swab to remove tobacco particles.

Aside from clogging during the unfiltered cigarette smoking treatment, some participants experienced melting of the plastic mouthpiece, which altered the diameter of the device orifice. Melting of the mouthpiece occurred on four occasions when participants did not remove the unfiltered cigarettes from the device after their smoking session. We instructed participants to smoke the unfiltered cigarette only down to the cigarette’s brand name stamp to prevent mouthpiece melting, and to remove the unfiltered cigarette when finished with smoking. Since the melting occurred after the four participants had finished smoking the unfiltered cigarette, it was unlikely that there were any unanticipated exposures due to the partially melted mouthpieces. These were replaced, and the problem was remedied through the additional advice provided to the study participants regarding unfiltered cigarette smoking. We routinely inspected the device at each visit and conducted thorough cleaning and maintenance during the wash-out period.

## 4. Discussion

We found that utilization of the CReSS device in a naturalistic study setting was feasible and had fairly high compliance rates. This is one of few studies to utilize the CReSS device to evaluate ST longitudinally, including multiple time point measurements under multiple treatment conditions (different types of cigarettes) and in the smokers’ natural environment. However, we also found that it is necessary to understand and plan for several practical challenges in using the CReSS in such a study.

First, research teams should be comfortable with device nuances and consider necessary preparatory steps prior to study implementation. These include standardized training for research staff in calibration, cleaning, and device testing. We found it necessary to be flexible in order to navigate device functioning and then to adjust the research plan and protocol [3,9]. We did not anticipate clogging or mouthpiece melting problems due to the use of unfiltered cigarettes since we found no other CReSS studies using unfiltered cigarettes. In retrospect, this does not seem too surprising. In fact, the original intended use for the cigarette filter was to prevent tobacco particles from entering smokers’ mouths. It was not until the “health scares” of the 1950s and 1960s that filters were marketed to convince smokers that filtered cigarettes were somehow “safer” [23]. Nevertheless, we were able to adapt our procedures, participant training, and study protocol in order to manage these relatively minor technical issues. This was a proof-of-principle study testing topography measurement in order to conduct a larger randomized trial of filtered vs. unfiltered cigarette use and exposures. 

Second, device maintenance and proper training of research staff can mitigate problems and facilitate troubleshooting to prevent malfunctioning. While our device cleaning practices were not standardized to manufacturer recommendations, we do not believe they altered device functioning; existing cleaning recommendations are based on clinical lab settings, not naturalistic settings as in our study [3]. For similar clinical trials, we suggest that protocols include the following: regular calibration; cleaning of device orifices, mouthpieces, and tubing; and consistent training for research staff and participants. We found it necessary to have a backup device available in case of malfunctions, such as the date/time stamp issue or clogging. In addition, having extra mouthpieces, battery covers, screws, and orifice rings on hand was useful, as these smaller parts were easily misplaced and/or broken while in the participant’s possession. We attempted to control for device variability by issuing the same device to each participant throughout the study, regardless of the study arm, but we had to adapt to device malfunction as the study progressed. 

Despite the challenges described above, this report extends our understanding of ST measurement technology beyond laboratory and clinical settings. The high compliance rate for using the CReSS device of 5+ days/week for up to 8 h suggests the feasibility of using the device in a longitudinal RCT. The ST and other data from this novel cross-over trial of unfiltered cigarettes will be reported elsewhere and will help us understand how the CReSS device may be used reliably in future studies [22].

## 5. Conclusions

CReSS applications for smokers in naturalistic settings can provide insights into multiple areas of tobacco regulatory science research. For example, such studies can reveal ST patterns and exposures for different types of cigarettes, assess the relationship between smoke toxicants and ST, evaluate the relationship between nicotine dependence indicators and ST, and provide background data that may be used for tobacco product regulatory policy [7,11,24]. The portability and non-invasiveness of the CReSS device in assessing ST and associated exposures in the participants’ natural environment offer an accurate representation of these behaviors outside of the laboratory [4,8]. The CReSS device permits the convenient time- and date-stamped recording of cigarettes smoked throughout the day and may allow the assessment of changes in ST under different product conditions. 

CReSS devices are expensive, and researchers should consider the technical challenges described herein and factor device ordering and production times into the study timeline and design. However, overall, we found that the CReSS device can be a useful tool to evaluate the potential impact of changes in tobacco product design through product regulation [24]. 

## Figures and Tables

**Table 1:** Study Participant Demographics and Baseline Characteristics.

Demographic/Characteristic	Total *n* = 43
	*n* (%)
Age, Mean (SD)	36.7 (9.9)
Sex	
Female	18 (41.9)
Male	25 (58.1)
Ethnicity	
Not Hispanic/Latino	35 (81.4)
Hispanic/Latino	8 (18.6)
Mexican	5 (11.6)
Puerto Rican	2 (4.7)
Another Hispanic/Latino origin	1 (2.3)
Race	
White	30 (69.8)
Multiple races	5 (11.6)
Asian American	3 (7.0)
African American	2 (4.7)
Native Hawaiian or Other Pacific Islander	0
American Indian or Alaska Native	0
Unknown	3 (7.0)
Education	
High school or below	11 (25.6)
Some college or technical school	21 (48.8)
College graduate or above	11 (25.6)
Income (in $1000s), Mean (SD)	38.7 (35.4)
# years smoked cigarettes, Mean (SD)	17.2 (9.6)
# days smoked in past 30 days, Mean (SD)	30.0 (0.0)
# cigarettes smoked per day, past 30 days, Mean (SD)	14.5 (6.7)

## Data Availability

The data presented in this study are available on request from the corresponding author. The data are not publicly available because it contains identifiable information.
